# Identification and Assessment of Toxic Substances in Environmental Justice Cases

**DOI:** 10.3390/toxics12120900

**Published:** 2024-12-11

**Authors:** Xiaowei Xu, Dapeng Zhang, Jun Zhang, Zehua Zhao, Jing Hua, Yi Wang, Houhu Zhang, Qi Yu

**Affiliations:** 1 Nanjing Institute of Environmental Science, Ministry of Ecology and Environment of China, Nanjing 210042, China; xuxiaowei@nies.org (X.X.); zhangdapeng@nies.org (D.Z.); zhangjun@nies.org (J.Z.); zhaozehua@nies.org (Z.Z.); huajing@nies.org (J.H.); zhh@nies.org (H.Z.); 2 Department of Environmental Science, School of Environmental Science and Engineering, Suzhou University of Science and Technology, Suzhou 215009, China; yuqi@usts.edu.cn

**Keywords:** environmental judicial cases, heavy metals, solid waste, toxic substances, risk assessment

## Abstract

This study assessed heavy metal contamination in industrial solid waste (S1, S2, S3, and S4) from the Yangtze River Delta region, employing nine risk assessment methods including total content indices (e.g., Igeo, CF) and speciation indices (e.g., ICF, GCF). Four types of industrial solid waste not classified as hazardous but containing heavy metals were analyzed. Key findings revealed significant variability in risk assessments based on chemical speciation versus total content. For example, while S1, S3, and S4 exceeded background levels, S4 showed higher mobility of Pb, Cr (VI), Cu, Ni, and As despite lower overall content. Elements like Cd and Cr (VI) exhibited discrepancies between total content and speciation-based assessments due to low background values and high toxicity. Multi-element indices (DC, RI) indicated higher pollution degrees compared to speciation indices (GCF, GRI). These results underscore the need for integrating multiple assessment methods to accurately evaluate environmental risks in judicial practices.

## 1. Introduction

Article 338 of the criminal law of China stipulates that the discharge, dumping, or disposal of radioactive waste, waste containing pathogens of infectious diseases, toxic substances, or other harmful substances constitutes the crime of environmental pollution. Accurately identifying and assessing the nature and quantity of pollutants is crucial in determining whether a crime has been committed as well as the severity of the offense. Therefore, it is essential to conduct scientific, objective, and accurate evaluations of pollutants involved in environmental cases. In 2016, the Supreme Court and the Supreme Procuratorate issued a legal interpretation that clarifies the criteria for conviction and sentencing criminal cases pertaining to environmental pollution. Understanding heavy metal content is crucial because it directly affects whether waste is classified as toxic under the law, impacting legal determinations of environmental pollution offenses. However, the level of heavy metal content in solid waste that classifies the waste as a heavy metal-containing contaminant and thus a toxic substance under subparagraph (3) of Article 15 of the interpretation remains unclear. Identifying solid waste as toxic whenever heavy metals are detected would broaden the scope of conviction under article 1(5). Some scholars argue that solid waste with low heavy metal content should not be considered toxic, as China permits the discharge of pollutants within prescribed standards [1]. Therefore, establishing unified pollutant evaluation criteria is essential for maintaining evidentiary rigor, facilitating relevant case trials and adjudications, and improving judicial impartiality [2,3].

China currently has a well-developed system for identifying hazardous wastes, which are considered to be toxic substances [4,5]. However, there is no standardized method for identifying solid wastes that contain heavy metals but are not considered hazardous. This lack of clarity has created uncertainty in environmental justice cases. To address this issue, scholars have proposed various methods to evaluate heavy metal contamination and ecological risks in soils and sediments, and these methods can also be applied to the identification of heavy metal-containing contaminants.

Commonly used methods for assessing heavy metal contamination include the Index of Geoaccumulation (Igeo) [6] and Contamination Factor (CF) [7], which are calculated based on heavy metal content, as well as the Individual Contamination Factor (ICF) [8], which is calculated based on species fraction. To evaluate the ecological risk posed by metals, the Potential Ecological Risk Factor (ER) [9], which is based on total metal content, and the Risk Assessment Code (RAC) [10], which is based on the ratio of exchangeable components, are commonly employed. The conventional methods for risk assessment are limited to evaluating the risks associated with individual metals and cannot assess the cumulative environmental risks posed by multiple metals. To address this limitation, scholars have proposed alternative multi-element assessment methods, such as the Risk Index (RI) [11] and Degree of Contamination (DC) [12], which are both based on total content index, as well as the Global Risk Index (GRI) [5] and Global Contamination Factor (GCF) [12], which are based on heavy metal fractions.

Toxic substance identification in domestic environmental justice cases is predominantly based on heavy metal content in solid waste. This approach stems from the identification of hazardous wastes, which requires a comprehensive investigation of heavy metal content in solid waste [13,14]. However, it is now widely acknowledged that the mobility, bioavailability, and ecotoxicity of heavy metals are primarily dependent on their chemical speciation rather than on total content [2,9]. Consequently, heavy metal speciation-based methods have gained increasing attention.

In this study, we evaluated pollution and ecological risks of seven heavy metals (mercury (Hg), lead (Pb), chromium (Cr (VI)), copper (Cu), nickel (Ni), cadmium (Cd), and arsenic (As)) using five content indices (Igeo, CF, DC, ER, and RI) and four speciation indices (ICF, GCF, GRI, and RAC). The correlation between these two common index methods was assessed. Our findings provide guidance for identifying heavy metal-containing contaminants in legal proceedings, thereby enhancing the accuracy and reliability of legal determinations regarding environmental pollution. The aim of this study is to bridge the gap between scientific evaluation methods and legal applications by providing a robust framework for assessing heavy metal contamination in solid waste. By integrating advanced speciation analysis with established content indices, our research addresses the current ambiguity in defining toxic substances under Article 338, contributing to more precise and fair judicial decisions in environmental pollution cases.

## 2. Materials and Methods

### 2.1. Study Area

In this study, we examined four types of industrial solid waste (JSW) containing heavy metals in the Yangtze River Delta region, none of which met the criteria for hazardous waste identification. This area is a manufacturing hub that has increased in environmental pollution cases related to illegal solid waste transfer and disposal as China strengthens its enforcement efforts. For each type of solid waste, five samples were collected to ensure comprehensive representation; these samples originated from land reclamation and construction projects where the waste was used as substitutes for materials such as earth, sand, and stone. The sampling locations for the four types of solid waste, along with their respective geographic coordinates, are: S1: (120.541223175° E, 31.497959810° N); S2: (119.441978819° E, 31.743864732° N); S3: (120.174468643° E, 32.538249211° N); S4: (118.426719792° E, 33.637568670° N).

### 2.2. Procedure for the Sequential Extraction of Heavy Metal Species

The solid waste samples used in this study were from land reclamation and construction land leveling projects that used the waste as a substitute for production materials such as earth, sand, and stone. The solid waste was used to fill underground goafs, open pit mining surface excavation areas, soil extraction sites, underground mining subsidence areas, and natural pits and hollows. The seven specific heavy metals—mercury (Hg), lead (Pb), hexavalent chromium (Cr (VI)), copper (Cu), nickel (Ni), cadmium (Cd), and arsenic (As)—were selected due to their prevalence in industrial waste, high toxicity levels, and relevance in environmental legal cases. These metals are common in industrial solid waste because of their widespread use in manufacturing processes and are key pollutants under the GB 36600-2018 [15] standard. The soil environmental quality standard (GB 36600-2018) was chosen for its comprehensive criteria for assessing heavy metal contamination in soils, essential for environmental justice cases. This standard provides screening values for pollutants, including heavy metals, to determine ecological and health risks. Adherence to GB 36600-2018 ensures our analysis aligns with national regulations, enhancing result reliability and comparability by providing threshold values that distinguish between acceptable and harmful levels of heavy metals in solid waste.

We used the five-step sequential extraction procedure proposed by Tessier et al. [16]. The sequential extraction method is widely used to fractionate metals in environmental samples into five operationally defined fractions: exchangeable fraction extracted using 1 M MgCl_2_ and 0.5 M NH_4_Ac at pH 7, which includes metals loosely bound to soil particles; carbonate-bound fraction extracted using 1 M NaOAc at pH 5, which includes metals associated with carbonates; Fe-Mn oxide-bound fraction extracted using 0.04 M hydroxylamine-HCl in 25% acetic acid at pH 2, which includes metals adsorbed to iron and manganese oxides; organic-bound fraction extracted using 30% H_2_O_2_ and 1 M ammonium acetate at 85 °C, which includes metals bound to organic matter; and residual fraction obtained by dissolving the remaining sample in aqua regia, which includes metals tightly bound within mineral structures.

### 2.3. Analysis and Quality Control of the Data

The pretreatment and detection of all samples were carried out in the CMA certified laboratory of Nanjing Institute of Environmental Science, Ministry of Ecology and Environment (Nanjing, China). According to “Solid Waste–Determination of Mercury, Arsenic, Selenium, Bismuth, Antimony–Microwave Dissolution/Atomic Fluorescence Spectrometry” (HJ 702-2014) [17], the contents of Hg and As were measured by atomic fluorescence spectrometry, and the detection limits were 0.002 and 0.01 mg/kg, respectively. According to “Solid waste–Determination of 22 metal elements–Inductively coupled plasma optical emission spectrometry” (HJ 781-2016) [18], the contents of Pb, Ni, Cu, and Cd were measured by emission spectrometry, and the detection limits were 1.4, 0.4, 0.5, and 0.01 mg/kg, respectively. According to “Solid waste–Determination of Hexavalent Chromium–Alkaline digestion/flame atomic absorption spectrophotometric” (HJ 687-2014) [19], the content of Cr (VI) was measured by flame atomic absorption spectrophotometry, and the detection limit was 0.5 mg/kg. The accuracy of the methods was routinely assessed according to HJ/T 166-2004 [20], the Technical Specification for Soil Environmental Monitoring. Metal contents were found to fall within 81–109% of the certified values, a range considered acceptable under this standard. This ensures the reliability of the results by accounting for potential variability in sample preparation and analysis.. Three replicates were performed to determine both total and fractionated metal contents. Additionally, a Shapiro-Wilk test was conducted for all metal species of interest, which passed at a 0.05 significance level.

### 2.4. Content and Speciation Indices

We used nine indicators to assess pollution and ecological risk based on the physical characteristics and total content of metal elements in the samples. Table 1 provides a detailed description of these indicators, which encompass morphological/total content and individual/multi-element analysis methods and are widely used for characterizing metal pollution and ecological risks. To ensure comparability, the results for the nine indicators were evaluated using four levels of pollution or ecological risk: low, medium, equivalent, and high.

Kwon and Lee [21] suggested two methods to ensure consistency of background values for different analytical techniques. The first method involves determining a general geological reference value and applying it to various methods. Alternatively, one can establish the reference value of the pre-industrial or pre-civilization era. In this study, we adopted the second approach and used exposed soil around a solid waste landfill as the background sample, with the average content of Hg, Pb, Cr (VI), Cu, Ni, Cd, and As being 0.057, 31, 0.5, 22, 23, 0.18, and 5.25 mg/kg, respectively. To evaluate the ecological risk of metals, calculating the toxic reaction factors for each metal is crucial. We used the toxic factors proposed by Hakanson [22], which were 40, 5, 40, 5, 30, and 10 for Hg, Pb, Cr (VI), Cu, Cd, and As, respectively. However, no toxic reaction factor for Ni was provided in their study. Because Ni and Pb are in the same column on the periodic table and are expected to have similar impacts on the environment, we used the toxic reaction factor of 5 for both Ni and Pb.

## 3. Results and Discussion

### 3.1. Total Contents and Speciation of Heavy Metals in the Solid Waste Samples

Table 2 demonstrates that the average content of heavy metals in the four types of solid waste exceeded their respective background levels. This suggested that the unregulated disposal of these wastes, without any environmental protection measures, could have adverse effects on the surrounding environment. In legal proceedings, these four types of solid waste are classified as toxic substances, and the penalty severity is determined by the quantity of waste involved. The contents of Pb, Ni, and As in solid waste S1 exceeded the screening value of environmental quality standards for soils in China (GB 36600-2018) by 1.7, 2.5, and 3.9 times, respectively, while the contents of Hg, Cr (VI), Cu, and Cd were below their corresponding screening values. Solid wastes S2 and S4 contained heavy metals within the screening value limits. In solid waste S2, the contents of Pb, Cr (VI), Cu, Ni, Cd, and As were lower than those in S4, whereas that of Hg was slightly higher in S2. The Cr (VI) content in solid waste S3 was 7.54 times higher than the screening value, while the contents of the other heavy metals were below their corresponding screening values.

**Figure 1:**
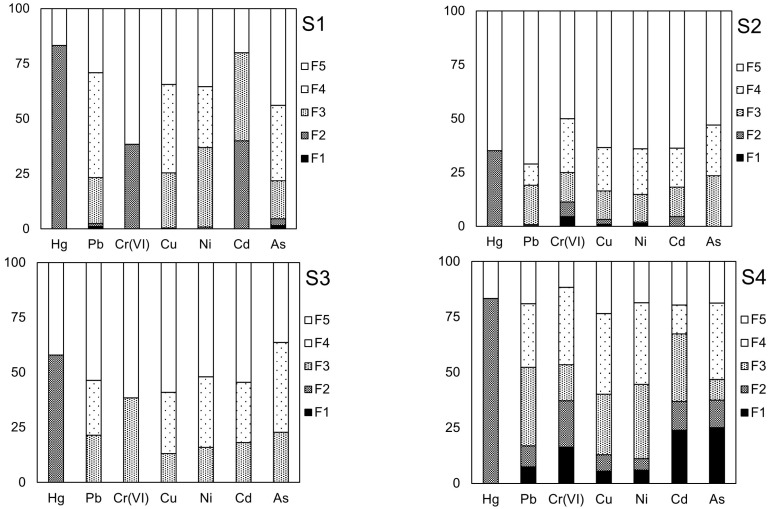
Speciation of metals in solid wastes (%).

Table 2 also displays the contents of heavy metals in the individual fractions of the leaching solution from the four solid wastes, and Figure 1 shows their chemical speciation as a percentage of the total content. The accuracy of the BCR sequential extraction procedure was verified using recovery rates (R; %) for Pb, Cr (VI), Cu, Ni, Cd, and As, which ranged from 82.4 to 159.3%. Other studies have reported similar results, with recovery rates ranging from 90.3 to 130.9% [23] and 72.0 to 123.9% [24]. However, the recovery rate for Hg was low, ranging from 39.2 to 75.0%. This suggested that the determination of Hg chemical speciation in solid waste using the BCR continuous extraction method may be unreliable, as each extraction step can affect Hg volatilization [25]. Further research is needed to clarify this issue.

The solid wastes studied generally exhibited higher contents of selected heavy metals bound to immobile fractions (F4 and F5), except for S4, in which the mobile fractions (F1 + F2 + F3) of heavy metals were close to or even higher than the immobile fraction content. The fractions could be classified into three categories based on their toxicity: F1 and F2 fractions contain metals that are directly toxic; F3 and F4 fractions consist of potentially toxic members that can be easily degraded; and the F5 fraction is considered to be non-toxic [16]. In S4, the mobile fractions of Hg, Pb, Cr (VI), Cu, Ni, Cd, and As accounted for 83.33%, 52.20%, 53.49%, 40.12%, 44.64%, 67.39%, and 46.88%, respectively, indicating that rainwater leaching could transfer these heavy metals to the surrounding environment. The mobile fractions of heavy metals in the other solid waste samples were generally below 20%. Moreover, the distribution of heavy metals in mobile fractions of S1, S2, S3, and S4 was as follows: Hg > Cd > Cr (VI) > Ni > Cu > Pb > As (S1), Hg > Cr (VI) > As > Pb > Cd > Ni > Cu (S2), Hg > Cr (VI) > As > Pb > Cd > Cu > Ni (S3), and Hg > Cd > Pb > Cr (VI) > As > Ni > Cu (S4). These findings have important implications for environmental justice cases. Specifically, these results confirmed that identifying toxic substances based solely on total heavy metal content in solid waste was insufficient; instead, knowledge of the chemical forms of these substances should be the primary criterion.

### 3.2. Risk Assessment of Selected Heavy Metals in Solid Wastes

#### 3.2.1. Risk Assessment of Each Individual Metal

Table 3 presents the assessment results for single heavy metal contamination and ecological risk in solid waste. For solid waste S1, Pb, Ni, and As significantly exceeded the screening value, indicating their toxic properties. The degree of heavy metal contamination based on the total content index was generally higher than that based on the speciation index, except for Hg and Cd, which could be attributed to the speciation characteristics of heavy metals. Furthermore, the degree of contamination calculated by CF was higher than that calculated by Igeo in the total index, possibly due to the introduction of a constant (1.5) in the Igeo model to mitigate change of the background value due to lithogenic factors. The ecological risk posed by heavy metals is typically higher when assessed using ER compared to RAC. Although the levels of Hg and Cd were relatively low, trace amounts of F2, F3, and F5 components in these metals led to abnormal conclusions in the evaluation of morphological factors. Specifically, while the content of Hg was lower than the background value, RAC results identified it as posing medium to high risk. This discrepancy may be partially explained by differences in the definitions of the indices used for assessment. The RAC index utilizes the exchangeable fraction of metals to indicate the proportion of metals in solid waste that can be easily released into the environment [7,9]. However, the current form factor method had limitations and requires further optimization before it could be effectively applied for identifying toxic substances in legal proceedings.

The heavy metal content in solid waste S2 was below the screening value, and no heavy metals were detected in the F1 and F2 components, indicating a low overall risk. However, S2 has been identified as a toxic substance in judicial practice, which warrants further discussion. Using the form factor methods ICF and RAC, S2 was assessed as having low to medium ecological and environmental risk, provided that it was promptly cleaned and disposed of. From an enforcement perspective, it may be reasonable to classify solid waste S2 as a toxic substance in order to crack-down on environmental violations. However, from an objective assessment of environmental risks, there was a risk of over-criminalization by treating S2 and S1 equally. The values of total amount factors Igeo, CF, and ER of solid waste S2 generally indicated medium to low risk, with the exception of Cd and Cr (VI). Although the levels of Cd and Cr (VI) in S2 were below the screening value, the application of CF and ER resulted in Cd being considered high risk, and the application of CF and ER classified Cr (VI) as being medium to high risk. This results was mainly due to the extremely low environmental background values and high toxicity of Cd and Cr (VI). These findings showed that the total amount factor had limitations and requires further optimization for use in identifying toxic substances in judicial practice.

The heavy metal contents in solid waste S3 were significantly higher than the background value but remained within the screening value. The assessment of pollution degree and ecological risk for individual heavy metals indicated medium to high risk based on total amount factors Igeo, CF, and ER, and the values were notably higher than those obtained using morphological factors ICF and RAC.

Compared to solid waste S3, solid waste S4 contained lower levels of various heavy metals, but Pb, Cr (VI), Cu, Ni, and As were more available. The form factor ICF assessment results for a single heavy metal indicated medium-high and high risks, and they were significantly higher than those obtained from total factor Igeo and CF assessments. As these informal landfill solid wastes will be cleaned and disposed of, their impact on soil and groundwater in the external environment will mainly depend on the migration property and toxicity of heavy metals. The higher migration property of Pb, Cr (VI), Cu, Ni, and As in solid waste S4 resulted in a greater overall risk compared to solid waste S3, despite the fact that S4 generally had lower heavy metal content. This pattern was also observed in the results of both the ecological risk and pollution degree of solid waste S4. These results illustrated that relying solely on total amount to identify toxic substances resulted in inaccurate assessments of environmental risks.

#### 3.2.2. Risk Assessment of Multiple Heavy Metals in Solid Wastes

Multi-element indices are commonly used to assess the overall environmental impact of specific samples by integrating the risk levels of individual metal species [17]. Table 4 presents the results of the assessment of the comprehensive pollution degree and ecological risk of heavy metals in the solid waste samples using two approaches: form factor methods (GCF and GRI) and total factor methods (DC and RI). DC and RI analysis classified the comprehensive pollution degree and ecological risk of heavy metals in solid waste as being at the highest level (IV). In contrast, the GCF and GRI methods indicated that the pollution degree and ecological risk were between levels I and IV, which were significantly lower than the evaluation results of DC and RI. The form factor methods typically yield lower values than the total factor methods. Additionally, the GCF assessment level is often lower than that of GRI when assessing the comprehensive pollution degree and ecological risk of heavy metals. This discrepancy is due to the inclusion of toxicity response factors in GRI evaluations, which enhances the accuracy of the results. Scholars argue that greater attention should be devoted to the harmful effects of specific pollutants, as relying solely on comprehensive indicators may obscure the risks associated with individual pollutants [26].

### 3.3. Correlations Between the Total Content and the Speciation Index Approaches

**Figure 2:**
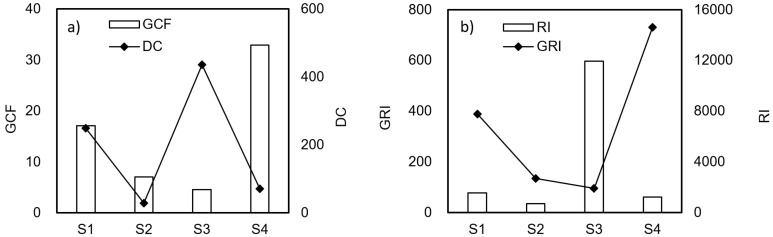
Correlations of assessment models of degree of multi-metal pollution and ecological risk.

Figure 2a illustrates the correlations between the contamination indices CGF and DC, and Figure 2b displays the correlations between the ecological risk indices RI and GRI. The bar chart in each figure represents the speciation index approaches, and the line indicates the total content index values. Results indicated a negative correlation between DC and GCF when applied to the comprehensive assessment of heavy metal contamination in solid waste. Similarly, GRI and RI exhibited a low negative correlation when applied to the comprehensive ecological risk assessment of heavy metals in solid waste. These results differed from those reported by Fei et al. [27] due to differences in sample types. They studied sediment from the same water area, where heavy metal content and morphology showed a positive correlation between total amount and morphological index at each point. However, the solid waste sources analyzed in our study contained different types and forms of heavy metals, leading to significant differences. According to some scholars, evaluating the environmental risks associated with heavy metals in solid waste can be achieved by prioritizing the collection of soil or groundwater samples from the surrounding areas. The resulting environmental impacts can then serve as a basis for judicial appraisal [3,26]. Our results indicated that both the total factor method and morphological factor method had limitations in assessing comprehensive pollution and ecological risk of heavy metals in solid waste, and further research is necessary to develop evaluation indexes for both methods.

## 4. Conclusions

This study explored methods for identifying and assessing heavy metal contamination in industrial solid waste from the Yangtze River Delta region, aiming to support environmental justice cases. By analyzing four types of JSW not classified as hazardous but containing heavy metals, we employed nine risk assessment methods, including total content indices (e.g., Igeo, CF) and speciation indices (e.g., ICF, GCF). These methods comprehensively reflected the enrichment effect, bioavailability, and toxicity of heavy metals. Key findings indicated that relying solely on total heavy metal content can lead to inaccurate risk assessments. For example, while S1, S3, and S4 had higher heavy metal contents than background levels, their risks varied significantly based on chemical speciation. In S4, despite lower overall content, Pb, Cr (VI), Cu, Ni, and As were more mobile, posing greater risks. Elements like Cd and Cr (VI) showed discrepancies between total content and speciation-based assessments due to their low background values and high toxicity. Multi-element comprehensive indices (DC, RI) generally indicated higher pollution degrees and ecological risks compared to speciation indices (GCF, GRI), which provided more conservative evaluations. The correlation between these models varied by sample type and heavy metal forms. Our results emphasize the importance of integrating multiple assessment methods and focusing on specific chemical forms of pollutants to ensure accurate evidence and fair sentencing in judicial practice. This research provides crucial support for improving environmental justice in China.

## Figures and Tables

**Table 1:** Indices used for risk assessment.

Index	Equation	Category	Description and Abbreviations
Index of geoaccumulation (Igeo): Contamination of single-metal (total content index)	$I_{geo}={log}_{2}\frac{C_{n}}{1.5B_{n}}$ $C_{n}$ is the measured content of metal n, $B_{n}$ is the background value, constant 1.5 is used to analyze the natural fluctuations and very small anthropogenic influences.	Igeo ≤ 1	Low (I)
		1 < Igeo ≤ 3	Moderate (II)
		3 < Igeo ≤ 5	Considerable (III)
		5 < Igeo	High (IV)
Contamination factor (CF): Contamination of Single-metal (total content index)	$CF=\frac{C_{n}}{B_{n}}$ $C_{n}$ is the measured content of metal n, $B_{n}$ is the background value.	CF ≤ 1	Low (I)
		1 < CF ≤ 3	Moderate (II)
		3 < CF ≤ 6	Considerable (III)
		6 < CF	High (IV)
Individual contamination factors (ICF): Contamination of Single-metal (speciation index)	$ICF=\frac{EXC+CARB+RO+OM}{RES}$ ICF for the various samples are obtained by dividing the sum of the four fractions (exchangeable, carbonate, reducible oxides, organic matter) by the residual fraction.	ICF ≤ 1	Low (I)
		1 < ICF ≤ 3	Moderate (II)
		3 < ICF ≤ 6	Considerable (III)
		6 < ICF	High (IV)
Degree of contamination (DC): Contamination of Multi-metal (total content index)	DC=∑CF CF is the contamination factor of metals.	DC ≤ 6	Low (I)
		6 < DC ≤ 12	Moderate (II)
		12 < DC ≤ 24	Considerable (III)
		24 < DC	High (IV)
Global contamination factor (GCF): Contamination of Multi-metal (speciation index)	GCF=∑ICF ICF is the individual contamination factor of metals.	GCF ≤ 6	Low (I)
		6 < GCF ≤ 12	Moderate (II)
		12 < GCF ≤ 24	Considerable (III)
		24 < GCF	High (IV)
Potential ecological risk factor (ER): Ecological Risk of Single-metal (total content index)	$ER=T_{r}\times CF$ $T_{r}$ is the toxic response factor of elements. CF is the contamination factor of elements.	ER ≤ 40	Low (I)
		40 < ER ≤ 80	Moderate (II)
		80 < ER ≤ 160	Considerable (III)
		160 < ER	High (IV)
Risk assessment code (RAC): Ecological Risk of Single-metal (speciation index)	RAC=EXC%+CARB% EXC% and CARB% are percentages of metals in exchangeable and carbonate fractions.	RAC ≤ 10%	Low (I)
		10% < RAC ≤ 30%	Moderate (II)
		30% < RAC ≤ 50%	Considerable (III)
		50% < RAC	High (IV)
Risk index (RI): Ecological Risk of Multi-metal (total content index)	RI=∑ER ER is potential ecological risk factor of metals.	RI ≤ 150	Low (I)
		150 < RI ≤ 300	Moderate (II)
		300 < RI ≤ 600	Considerable (III)
		600 < RI	High (IV)
Global risk index (GRI): Ecological Risk of Multi-metal (speciation index)	$GRI=\sum ICF\times T_{r}$ ICF is individual contamination factor of elements.	GRI ≤ 150	Low (I)
		150 < GRI ≤ 300	Moderate (II)
		300 < GRI ≤ 600	Considerable (III)
		600 < GRI	High (IV)

**Table 2:** Total contents and the speciation of heavy metals in solid wastes (mg·kg^–1^).

		Hg	Pb	Cr (VI)	Cu	Ni	Cd	As
S1	Total	0.016	1340	1.1	1260	2260	0.05	237
	F1	ND	13.0	ND	4.10	ND	ND	4.40
	F2	0.010	15.0	0.5	3.30	18.1	0.02	8.20
	F3	ND	242	ND	335	684	0.02	44.0
	F4	ND	552	ND	537	522	ND	87.2
	F5	0.0020	337	0.8	460	673	0.01	112
	R, %	0.75	0.83	1.2	1.06	0.84	1.00	1.08
S2	Total	0.097	34.0	1.5	53.0	28.0	2.60	21.1
	F1	ND	ND	ND	ND	ND	ND	ND
	F2	0.022	ND	ND	ND	ND	ND	ND
	F3	ND	6.10	0.5	8.10	4.00	0.40	5.30
	F4	ND	7.00	ND	17.0	8.00	0.60	9.29
	F5	0.016	15.2	0.8	36.0	13.0	1.20	8.42
	R, %	0.39	0.82	0.87	1.15	0.89	0.85	1.05
S3	Total	0.12	540	43	668	693	48.1	18.1
	F1	ND	1.10	2.3	6.20	9.10	ND	ND
	F2	0.027	3.00	3.0	12.0	3.20	2.04	ND
	F3	ND	84.2	6.1	75.0	77.0	6.20	4.30
	F4	ND	45.0	11	114	127	8.12	4.21
	F5	0.050	327	22	359	384	28.5	9.39
	R, %	0.64	0.85	1.0	0.85	0.87	0.92	0.94
S4	Total	0.090	228	2.7	115	480	4.40	29.3
	F1	ND	22.0	0.7	9.30	30.4	1.14	8.21
	F2	0.030	28.2	0.9	12.1	26.2	0.60	4.05
	F3	ND	104	0.7	44.0	169	1.40	3.07
	F4	ND	85.0	1.5	59.0	185	0.61	11.2
	F5	0.0060	56.2	0.5	38.0	94.4	0.93	6.44
	R, %	0.40	1.29	1.59	1.41	1.05	1.05	1.10
Background value	/	0.057	31.0	0.5	22.1	23.0	0.18	5.25
Screening value	/	38	800	5.7	18,000	900	65	60
Toxic response factors	/	40	5	40	5	5	30	10

Note: F1 = exchangeable fraction; F2 = carbonate fraction; F3 = reducible fraction; F4 = organic fraction; F5 = residual fraction. R = heavy metal recovery rate (%); ND = not detected.

**Table 3:** Degree of contamination and ecological risk of each heavy metal in different solid wastes.

		Contamination Degree						Ecological Risk			
		Speciation Index		Total Content Index				Speciation Index		Total Content Index	
		ICF	Class	I_geo_	Class	CF	Class	RAC	Class	ER	Class
S1	Hg	5.00	III	−2.42	I	0.28	I	0.83	IV	11.23	I
	Pb	2.44	II	4.91	III	45.10	IV	0.02	I	225.48	IV
	Cr (VI)	0.63	I	0.55	I	2.20	II	0.38	III	88.00	III
	Cu	1.91	II	5.25	IV	57.27	IV	0.01	I	286.36	IV
	Ni	1.82	II	6.04	IV	98.48	IV	0.01	I	492.39	IV
	Cd	4.00	III	−2.43	I	0.28	I	0.40	III	8.33	I
	As	1.28	II	4.91	III	45.14	IV	0.05	I	451.43	IV
S2	Hg	1.38	II	0.18	I	1.70	II	0.58	IV	68.07	II
	Pb	0.87	I	−0.45	I	1.10	II	0.00	I	5.48	I
	Cr (VI)	0.63	I	1.00	II	3.00	III	0.00	I	120.00	III
	Cu	0.69	I	0.68	I	2.41	II	0.00	I	12.05	I
	Ni	0.92	I	−0.30	I	1.22	II	0.00	I	6.09	I
	Cd	0.83	I	3.27	III	14.44	IV	0.00	I	433.33	IV
	As	1.75	II	1.42	II	4.00	III	0.00	I	40.00	II
S3	Hg	0.54	I	0.49	I	2.11	II	0.35	III	84.21	III
	Pb	0.41	I	3.54	III	17.42	IV	0.01	I	87.10	III
	Cr (VI)	1.00	II	5.84	IV	86.00	IV	0.11	II	3440.00	IV
	Cu	0.58	I	4.34	III	30.36	IV	0.03	I	151.82	III
	Ni	0.56	I	4.33	III	30.13	IV	0.02	I	150.65	III
	Cd	0.57	I	7.47	IV	266.67	IV	0.05	I	8000.00	IV
	As	0.89	I	1.19	II	3.43	III	0.00	I	34.29	I
S4	Hg	5.00	III	0.07	I	1.58	II	0.83	IV	63.16	II
	Pb	4.27	III	2.29	II	7.35	IV	0.17	II	36.77	I
	Cr (VI)	7.60	IV	1.85	II	5.40	III	0.37	III	216.00	IV
	Cu	3.26	III	1.80	II	5.23	III	0.13	II	26.14	I
	Ni	4.36	III	3.80	III	20.87	IV	0.11	II	104.35	III
	Cd	4.11	III	4.03	III	24.44	IV	0.37	III	733.33	IV
	As	4.33	III	1.88	II	5.52	III	0.38	III	55.24	II

**Table 4:** Degree of contamination and ecological risk of solid wastes.

	Contamination				Ecological Risk			
	Speciation Index		Total Content Index		Speciation Index		Total Content Index	
	GCF	Degree	DC	Degree	GRI	Degree	RI	Degree
S1	17.07	III	248.75	IV	388.61	III	1563.23	IV
S2	7.07	II	27.87	IV	134.92	I	685.02	IV
S3	4.55	I	436.11	IV	95.36	I	11,948.06	IV
S4	32.94	IV	70.40	IV	730.13	IV	1234.99	IV

## Data Availability

Data will be available once requested.
